# New Reports on the Portuguese Endemic Species, *Santolina impressa*: Secretory Structures, Essential Oil Composition and Antiviral Activity

**DOI:** 10.3390/plants12132391

**Published:** 2023-06-21

**Authors:** Ana Margarida Rodrigues, Ana Rita Mendes, Maria Filomena Caeiro, Ana Cristina Figueiredo, Lia Ascensão

**Affiliations:** Centro de Estudos do Ambiente e do Mar (CESAM Lisboa), Faculdade de Ciências da Universidade de Lisboa (FCUL), DBV, C2, Campo Grande, 1749-016 Lisboa, Portugal; amargaridacrodrigues@gmail.com (A.M.R.); ana.r.m.mendes@hotmail.com (A.R.M.); mfcaeiro@fc.ul.pt (M.F.C.)

**Keywords:** Asteraceae, biological activities, secretory structures, histochemistry, secondary metabolites, essential oil, herpes viruses, HSV-1, HSV-2

## Abstract

*Santolina impressa* is an aromatic Asteraceae species endemic to Portugal, traditionally used for its anti-inflammatory properties. The aim of this study was to characterize *S. impressa* secretory structures, analyze the essential oil (EO) from the aerial organs, and evaluate its antiviral activity against herpes simplex viruses HSV-1 and HSV-2. Secretory structures were investigated by light and scanning microscopy, and the secretion was histochemically characterized. The EO from the aerial organs in full blooming was analyzed by gas chromatography with flame ionization detection and gas chromatography–mass spectrometry. Antiviral assays were performed by direct contact with viral suspensions (virucidal effect), and in infected Vero E6 cells, at different time periods during the viral replication cycle. Two types of secretory structures were described, biseriate glandular trichomes and secretory ducts, producing an oleoresin and a resin rich in flavonoids, respectively. Fifty compounds were identified in *S. impressa* EO, accounting for 87% of the total constituents. Monoterpenes constituted the main EO fraction (82%), with β-pinene (13%) and β-phellandrene (10%) being their major components. The EO interacted with HSV-1 and HSV-2 in a dose-dependent manner, thereby inactivating both viral infections. The EO did not evidence a virucidal effect but inhibited the HSV-1 and HSV-2 infection in Vero cells in a dose-dependent manner. However, further studies are needed to investigate the mode of action in the replication cycle.

## 1. Introduction

The two human herpesviruses, known as type 1 and type 2 herpes simplex virus (HSV-1) and type 2 (HSV-2), typically cause mild diseases and may provoke severe infections in immunocompromised patients such as HIV seropositive and recipients of solid organ or bone marrow transplants [1]. At present, there are several systemic antiviral agents clinically used against herpesvirus, such as acyclovir and its prodrugs (valaciclovir, famciclovir, ganciclovir, ganciclovir, cidofovir, and foscarnet) approved by the US Food and Drug Administration (FDA) [2]. However, long-term prophylaxis and treatment with these drugs can result in the development of drug-resistance virus strains, especially in immunocompromised patients, where a prevalence of acyclovir-resistant viruses was greater than 3.5% [1].

The significant increase in microbiological strains resistant to the current clinical drugs led to an intense investigation of plants traditionally used in folk medicine to search for new bioactive compounds. In fact, aromatic and medicinal plants, used by humans for millennia, are a source of a great diversity of natural products, useful metabolites with medicinal value, that even today, continue to be essential for about 80% of the people in developing countries that have not access to Western Medicine [3]. Many of these pharmacologically active compounds are secondary metabolites that are bio-synthesized and accumulated in specialized secretory structures of numerous taxa. Vascular plant families such as Asteraceae, Lamiaceae, Apiaceae, Rosaceae, and Malvaceae, among others, are well known for their ability to produce phytochemicals with medicinal and industrial value [4].

*Santolina* L. is an Asteraceae genus (tribe Anthemideae) with remarkable taxonomic complexity, endemic to the Mediterranean Basin, comprising about 25 species of aromatic evergreen perennials and dwarf subshrubs widely distributed in southern Europe and northwest Africa [5]. Many *Santolina* species have been reported for their folk medicine traditional uses, namely in herbal remedies for the treatment of various disorders, as anti-inflammatory, antiseptic, antispasmodic, digestive, and analgesic agents [6]. Other species, particularly *S. chamaecyparissus*, the most widespread in the western Mediterranean region, are cultivated as ornamentals and used in landscape architecture or as sources of essential oils (EOs). In fact, due to their strong and agreeable fragrance and extensive ethnobotanical uses in Mediterranean countries, the essential oil composition of several *Santolina* species has been intensively investigated, as well as their biological activities [7,8,9,10,11,12,13,14,15].

**Figure 1:**
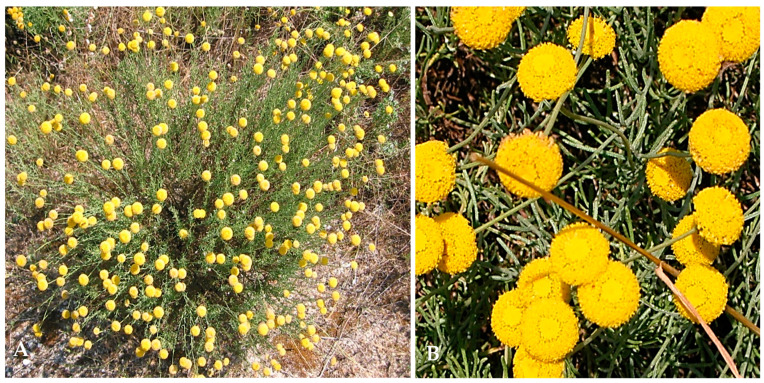
*Santolina impressa* in full blooming: (**A**) in its natural habitat; (**B**) detail of leaves and capitula.

*Santolina impressa* Hoffmanns. and Link is a Portuguese endemic species distributed along 1.022 km² in the south-eastern coastal area of Portugal from the estuary of the Sado River to Cape Sines [16,17,18]. This perennial shrub, with small and cylindrical grey-green leaves and stalked yellow capitula, grows in acidic sandy soils, stabilized dunes (often in palaeodunes), and sometimes under the cover of pine trees (Figure 1). 

In Portuguese traditional medicine, *S. impressa*, commonly named “macetão-das-areias”, is used for preparing herbal tisanes that are recognized for their anti-inflammatory properties in oropharyngeal infections and in several gastrointestinal ailments, namely in disturbed digestion, dyspepsia, and spasmodic abdominal pains [19,20]. Although morphological and phytochemical studies have been conducted on several *Santolina* species around the Mediterranean, little is known about *S. impressa.* Indeed, thus far, the few studies available have concentrated on its distribution and taxonomic characterization [16,21]. 

Recently, in an attempt to explain the traditional use of this species, the composition of the EO and its anti-inflammatory and antimicrobial properties were investigated, and a significant anti-inflammatory effect and antifungal activity against several dermatophytes were reported [14]. Likewise, the phenolic profile and biological activities of the decoctions of *S. impressa* were also studied. The results showed that these aqueous extracts are safe, non-toxic, and display a mild antimicrobial activity against the main microbial agents of dental caries, which supports its use as antiseptic mouthwash for the prevention of oral infectious diseases [22]. Despite these two studies, the biological activities of *S. impressa* are still poorly explored when compared to other *Santolina* species, and as far as we know, their secretory structures have not yet been investigated. 

This species is classified as Least Concern on the IUCN Red List of Threatened Species, and within its restricted range, the populations are considered abundant and stable [23]. The inclusion of these species on the IUCN Red List is mostly justified by the increasing influence of human activity due to urban and agricultural expansion and habitat destruction [21]. For conservation purposes and sustainable use due to their potential biotechnological applications, micropropagation of *Santolina* endemic species is a matter of increasing relevance [12,24,25].

*S. impressa* has been included in Annex II of the Habitats Directive (Council Directive 92/43/EEC) [26] since it is considered of community interest whose conservation requires the designation of special areas of conservation. Given community interest and to contribute to a better knowledge of this species, the present study aimed to characterize the secretory structures of the aerial organs of *S. impressa,* analyze the EO isolated during full blossom, and evaluate its antiviral activity against herpes simplex viruses HSV-1 and HSV-2.

## 2. Results and Discussion

### 2.1. Characterization of the Secretory Structures

#### 2.1.1. Surface Overview of Mature Leaves and Inflorescence Florets

**Figure 2:**
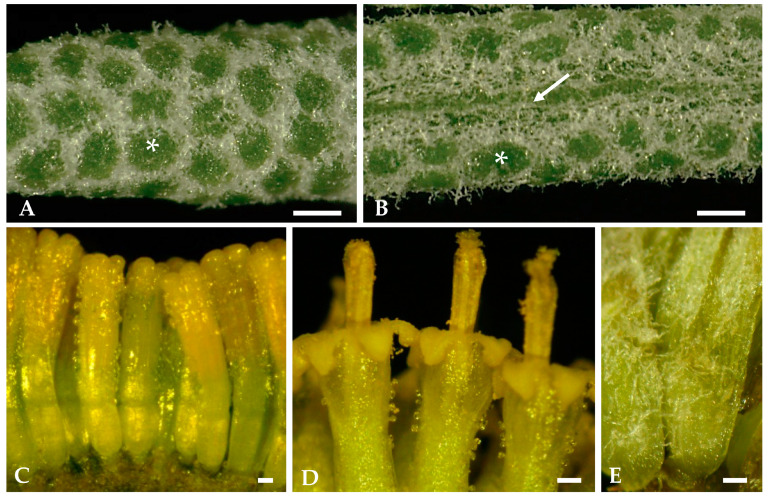
Stereomicrographs of *Santolina impressa* mature leaves (**A**,**B**), capitulum florets (**C**,**D**), and bracts (**E**): (**A**,**B**) Close-up of the dorsal and ventral surface of leaves in front view. A slit (arrow) is visible in the ventral surface, formed by the rolling of the leaf blade from dorsal to ventral surface. Green circular regions (asterisks) correspond to the top of closely adpressed leaf lobes. (**C**,**D**) Florets before and in anthesis, respectively, showing numerous glandular trichomes on the outer surface of the corolla tube. (**E**) The woolly tomentose indumentum of the interfloral bracts. Scale bars = 300 µm.

The small subcylindrical green-whitish leaves of *Santolina impressa*, when observed under a stereomicroscope, show that only their adaxial surfaces are visible since the leaf blade rolls from dorsal to ventral surface, leaving no more than a narrow slit between the two rolled leaf margins (Figure 2A,B). Long and flexuous hairs protrude through this slit (Figure 2B), covering it almost completely. On the surface of these apparently entire leaves, the green circular regions observed, surrounded by whitish hairs, correspond to the top of closely adpressed leaf lobes (Figure 2A,B), which is shown in detail in the following micrographs of leaf cross sections.

The hermaphroditic florets in the yellow monogamous capitulum show numerous glandular trichomes on the outer surface of the corolla, mainly located at the apical zone and at the corolla lobes (Figure 2C,D). Glandular trichomes are absent on the receptacle and ovary (Figure 2C), and a woolly tomentose indumentum, such as that present on the leaves, covers the outer surface of the interfloral bracts (Figure 2E).

#### 2.1.2. Distribution, Micromorphology, and Anatomy of Secretory Structures

The aerial organs of *S. impressa* bared a dense woolly-tomentose indumentum with two types of trichomes: (a) long, simple, uniseriate, and multicellular non-glandular trichomes; (b) multicellular biseriate glandular trichomes.

**Figure 3:**
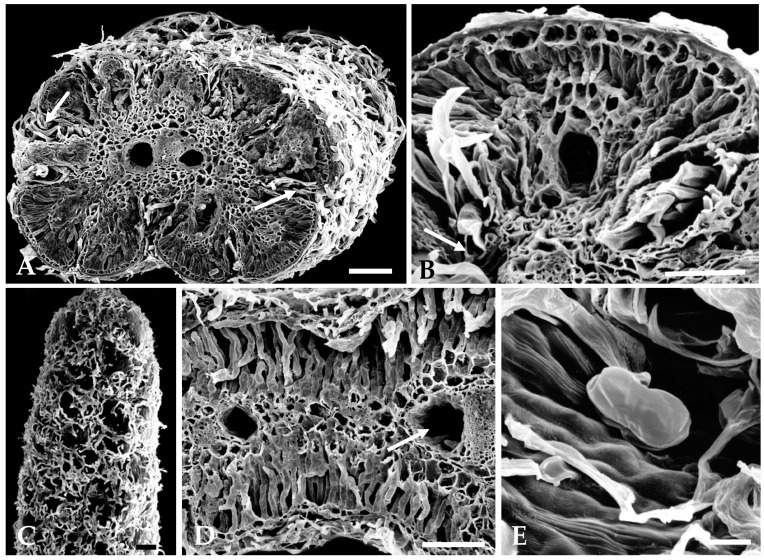
Scanning electron micrographs of leaves: (**A**) Cross section of the revolute and polylobed leaf showing the deep and narrow laciniae with numerous trichomes (arrows) and two secretory ducts, one of each side of the central vascular bundle. (**B**) Detail of a leaf lobe exhibiting a secretory duct. In the laciniae, non-glandular trichomes and one glandular trichome presenting cuticle rupture (arrow) are evident. (**C**) Top view of the leaf adaxial surface. Note the long and filamentous non-glandular trichomes that emerge from laciniae. (**D**) Portion of a leaf showing the compact mesophile and two secretory ducts, one in the leaf lobe and the other near the central vascular bundle (arrow). (**E**) A biseriate glandular trichome of the vesicular monotype. Scale bars = 150 µm (**A**–**D**), 25 µm (**E**).

On the revolute and deeply lobed small leaves, non-glandular trichomes are particularly concentrated in the laciniae, thin cavities formed between two successive leaf lobes that are well seen in transverse leaf sections (Figure 3A,B). Glandular trichomes are scattered among the long and filamentous non-glandular trichomes and are partially hidden by them (Figure 3A,B). Non-glandular trichomes, due to their great length, emerge from the laciniae and extend to the adaxial leaf surface, covering it almost completely (Figure 3A,C). They also protrude through the narrow slit formed between the two rolled-down leaf margins, filling it entirely (Figure 3A).

**Figure 4:**
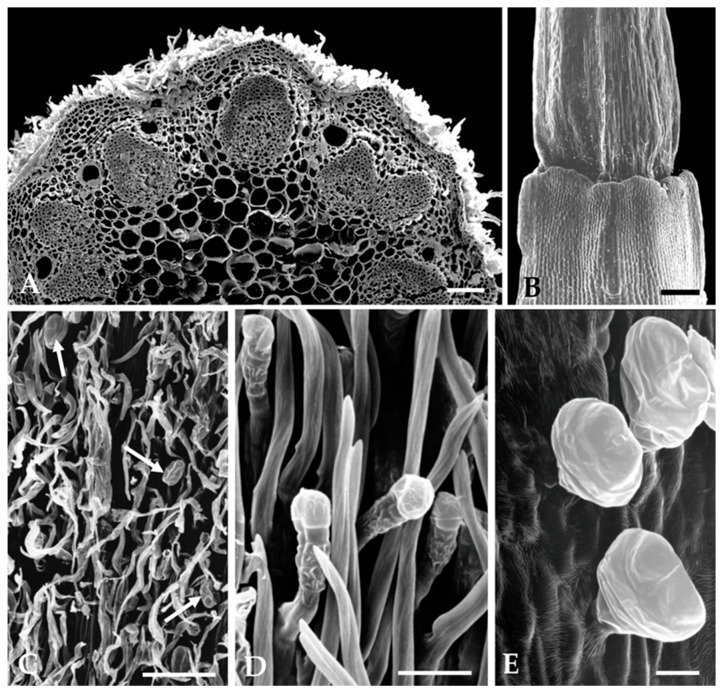
Scanning electron micrographs of floral organs: (**A**) Cross section of capitulum peduncle presenting numerous cortical secretory ducts adjacent to the vascular bundles. (**B**) Ovary and lower region of corolla tube devoid of trichomes. (**C**) Non-glandular trichomes densely packed, covering the glandular ones (arrows) on the capitulum peduncle surface. (**D**) Abaxial surface of the interfloral bracts showing the stipitate morphotype of glandular trichomes. (**E**) Biseriate glandular trichomes of vesicular morphotype on the outer epidermis of floret’s corolla tube. Scale bars = 150 µm (**A**–**C**), 25 µm (**D**,**E**).

Likewise, on the stem and floral peduncle, this mat of long and filamentous non-glandular trichomes is also present, partially concealing the glandular trichomes (Figure 4A,C). On the capitulum, glandular trichomes occur on the outer surface of the tubular corolla of florets, mainly at the apical zone (Figure 4E), and are absent on the inner surface of the floret’s corolla tube, the ovary, and receptacle (Figure 4B). On the involucral and interfloral bracts, glandular trichomes are located on the abaxial surface, dispersed among non-glandular trichomes (Figure 4D). 

The biseriate glandular trichomes, club-shaped, are formed by two basal cells embedded in the epidermis, two stalk cells and six head cells on vegetative organs and on the corolla of the capitulum florets (Figure 3E and Figure 4E), and ten head cells on the interfloral bracts. (Figure 4D). According to the recent classification of the leaf glandular trichomes in Asteraceae [27], the trichomes on the vegetative organs and floret’s corolla of *S. impressa* belong to the vesicular morphotype, characterized by its short biseriate base and body, while those on the interfloral bracts fit well in the stipitate morphotype characterized by an elongated and narrow biseriate body and a wider biseriate apex.

**Figure 5:**
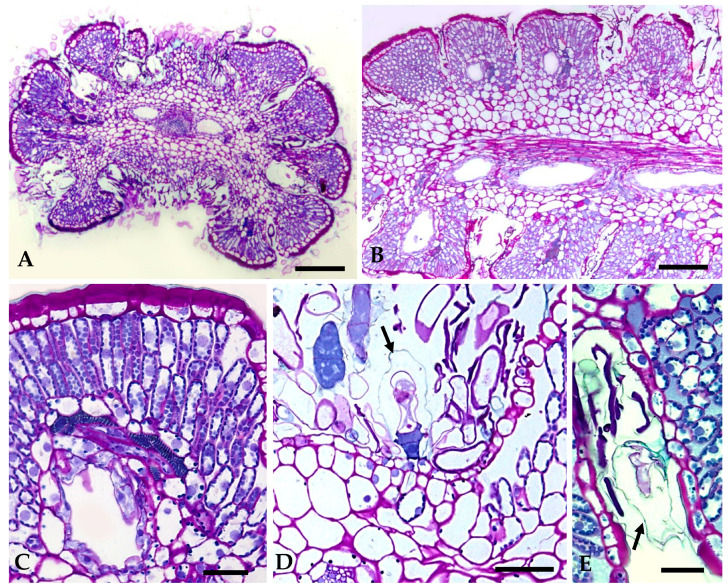
Light micrographs of historesin sections of fully expanded leaves stained with periodic acid—Schiff’s reagent/toluidine blue O: (**A**) Cross section of a leaf displaying its polylobed structure. Two secretory ducts, one on each side of the vascular bundle of the midrib, are clearly seen. (**B**) Paradermal section of a leaf showing duct distribution. One duct per leaf lobe and one of the two others that run parallel to the central vascular bundle is evident. (**C**) Detail of a leaf lobe presenting a duct with a large lumen. Note the thick cuticle of the epidermal cells at the top of the leaf’s rounded lobe. (**D**,**E**) High magnification of laciniae showing numerous non-glandular trichomes and glandular trichomes with large subcuticular spaces (arrows). Scale bars = 150 µm (**A**,**B**), 25 µm (**C**–**E**).

Secretion accumulates gradually in a subcuticular space formed by the detachment of the cuticle from the outer wall of the uppermost tier of head cells. However, in the trichome’s post-secretory phase of the vesicular morphotype, the cuticle is entirely detached along the outer wall of all secretory cells (Figure 5D,E, arrows). Secretion is released to the outside by cuticle rupture. 

Secretory ducts occur in the leaves, stems, and bracts. In leaves, one secretory duct occurs in the mesophyll per leaf lobe, and two ducts running parallel to the main vein, one on each side of the vascular bundle, are also observed (Figure 3A,B,D and Figure 5A,B). In the stem and in the peduncle of the inflorescence, the secretory ducts are numerous and placed in the cortical parenchyma adjacent to the vascular bundles (Figure 4A). On the bracts, only two central ducts are present.

In mature organs, the ducts have a large lumen, delimited by a single layer of flattened epithelial cells that are surrounded by a sheath of larger and thick-walled cells (Figure 5C).

#### 2.1.3. Histochemistry of Secreted Material

The results of the histochemical tests carried out to characterize the main classes of compounds present in the secreted material produced by the glandular trichomes and secretory ducts of *Santolina impressa* are summarized in Table 1.

**Figure 6:**
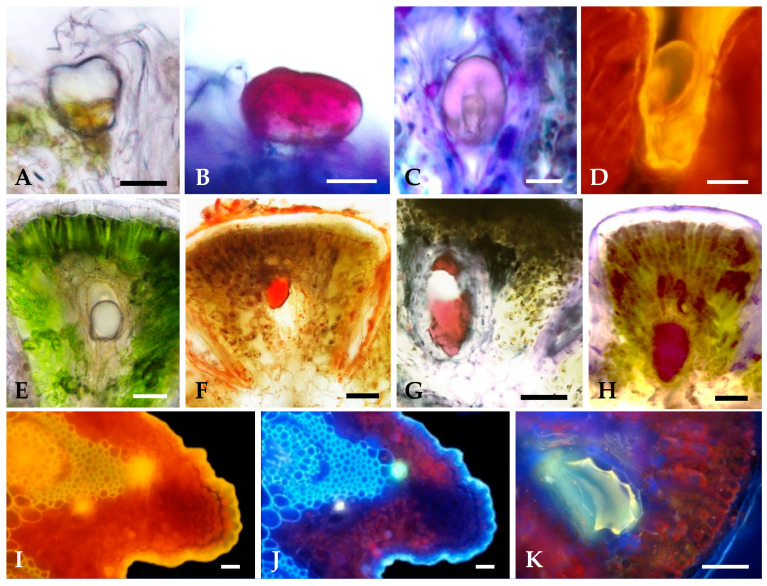
Histochemical characterization of the secretions of *S. impressa* glandular trichomes (**A**–**D**) and secretory ducts (**E**–**K**): (**A**) In vivo showing the large subcuticular space. (**B**) Nile Blue stained red the acidic lipids in the subcuticular space and in the glandular head cells. (**C**) Secretion, within the subcuticular space, stained violet with Nadi reagent. (**D**) Yellow autofluorescence observed under blue light. (**E**) In vivo showing the translucent secretion in the duct lumen. (**F**) Staining of total lipids with Sudan IV. (**G**) Acidic lipids stained red with Nile Blue A. (**H**) Nadi reagent stained dark red the secretion in the duct lumen. (**I**,**J**) Yellow and blue-light autofluorescence of duct secretion observed under blue and UV light, respectively. (**K**) Light yellow secondary fluorescence induced in the secretion by aluminum trichloride under UV light. Scale bars = 50 µm.

The translucent secretion produced by both secretory structures (Figure 6A,E) stained intensively in response to all lipophilic dyes used, namely, those to total lipids (Figure 6F), acid and neutral lipids (Figure 6B,G) and unsaturated lipids. The secreted material stored in the subcuticular space of the glandular trichomes stained violet with Nadi reagent (Figure 6C), indicating the presence of an oleoresin, whereas that accumulated in the lumen of the ducts stained dark red (Figure 6H), showing its resiniferous nature. The positive reaction to potassium dichromate and ferric trichloride demonstrated the presence of phenolic compounds, which is confirmed by the bright yellow secretion autofluorescence observed under blue light (Figure 6D,I) and blue autofluorescence under UV light (Figure 6J). This autofluorescence could be diagnostic for flavonoids; moreover, the light-yellow secondary fluorescence observed under UV after the application of aluminum chloride (Figure 6K) indicated the presence of flavonoids in the secreted material. Histochemical tests for the detection of hydrophilic substances (pectins and polysaccharides) were negative, showing that these classes of compounds are not present in the secretion of both glandular structures.

Secretory structures are frequent in all organs of Asteraceae species, although their main diversity is found in the leaves [28]. Secretory ducts and biseriate glandular trichomes of the vesicular morphotype are common in this family, especially in the Anthemideae tribe [27], and have been described for several species [29,30,31,32,33,34].

Biseriate glandular trichomes of the stipitate morphotype are not so frequent in An-themideae, but their occurrence has also been reported for several species [35,36,37]. In both glandular morphotypes, the release of the secreted material, stored in the trichome’s subcuticular space, occurs by cuticle disruption once the secretory process is finished. 

To our knowledge, this is the first time that the secretory structures of *S. impressa* have been studied, and the main classes of compounds present in their secreted material have been characterized histochemically. The micromorphology and distribution pattern of the secretory structures, as well as the major classes of components in the secretion of *S. impressa,* are similar to those described for *S. insularis* [38], *S. leucantha* [39], and *S. ligustica* [33]. The histochemical results indicate that trichomes synthesized an oleoresin, while ducts produce a resin, being both secretions rich in phenolic compounds. The presence of terpenoids (essential oils and resiniferous acids) and phenolic compounds in *S. impressa* are consistent with the phytochemical data available for this species [14,22,40].

The small, subcylindrical, and deeply lobed leaves of *S. impressa*, with marked revolute margins and woody indumentum, are morpho-anatomical leaf traits that can be interpreted as valuable adaptative strategies to the severe drought of the coastal sand dune habitat, where the specie grows. In fact, in these dune ecosystems, the summers are hot and dry with high temperatures and irradiance and low or no precipitation, and native plants have developed morpho-anatomical adaptations to survey these dry habitat conditions [41]. Following the same line of reasoning, the glandular trichomes concentrated in the laciniae could be a strategy to defend leaves from sandblasting, helping the glandular trichomes to maintain their functional integrity and the secreted material to protect the plant against biotic injuries, as microorganisms and herbivory.

### 2.2. Essential Oil Composition

The EO from the flowering aerial parts of *S. impressa* was obtained in a yield of 0.5% (v/f.w, volume/fresh weight) and characterized by a strong and pleasant chamomile-like fragrance and a faint yellow color. *S. impressa* EO chemical composition is detailed in Table 2, in accordance with their elution order on the DB-1 column. Fifty components were identified, accounting for 87% of the total EO. Monoterpenes constituted the major fraction of the EO (82%), with hydrocarbons and oxygen-containing compounds in high percentages (48% and 34%, respectively). Sesquiterpene hydrocarbons (2%) and oxygen-containing sesquiterpenes (2%) attained much lower percentages.

β-Pinene (13%) and β-phellandrene (10%) were the major components identified in *S. impressa* EO, followed by yomogi alcohol (9%), limonene (8%), camphor (7%), and 1,8-cineole and β-myrcene (both 6%). Qualitatively, the results obtained in the present study with the main EO components agree with a recent report on the EO chemical composition of *S. impressa,* also grown in Portugal [14]. The quantitative differences may be partly ascribed to several factors, including plant developmental stage; harvest time; and, most probably, diverse yearly climatic parameters [42].

The main constituents identified in the EO of *S. impressa* here studied have also been found in the EOs of other *Santolina* spp., namely in *S. chamaecyparissus*, the most widespread species. Although artemisia ketone is the dominant component of *S. chamaecyparissus* EOs isolated from specimens grown in several Mediterranean countries [8,43,44], high amounts of 1,8-cineole, β-myrcene, and β-phellandrene were also detected [11,45,46]. On the other hand, β-myrcene has also been identified as the major constituent of *S. ligustica* [47], *S. insularis* [48], and *S. corsica* [9] EOs. When comparing the EO composition of *S. impressa* with that reported for *S. rosmarinifolia* L. ssp. *rosmarinifolia* from the same aggregate [49,50,51], a higher similarity is recorded, which emphasizes the taxonomical proximity between the two species [16,17,21].

### 2.3. Cytotoxicity of Essential Oil 

**Figure 7:**
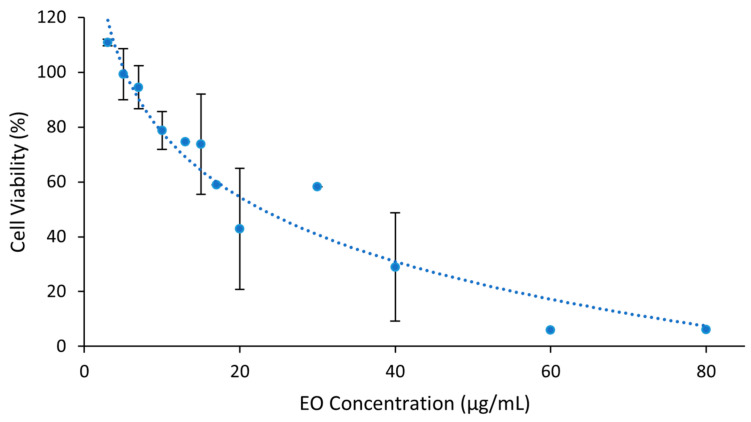
Cytotoxicity of the EO of *S. impressa* against Vero E6 cells (dots and blue trendline). Mean ± SD of at least two assays with four replicates for EO concentrations under 60 μg/mL.

Vero E6 cells’ susceptibility to *S. impressa* EO diluted in Medium-FBS2 was calculated by the thiazolyl tetrazolium bromide (MTT) assay. A dose-dependent cytotoxicity (complement of cell viability) was observed, with about 80% of cell viability for the EO at 10 μg/mL and a maximum non-cytotoxic concentration (MNCC) of 7.04 μg/mL (Figure 7). The concentration inducing 50% of toxicity (CC_50_) was 22.91 μg/mL. This CC_50_ value, <100 μg/mL, is indicative that *S. impressa* EO may be a promising natural product against infectious diseases [52].

### 2.4. Essential Oil Antiherpetic Activity 

#### 2.4.1. Virucidal Effect

In all assays carried out in the present study, *S. impressa* EO has not demonstrated a virucidal effect against both viruses, even when assayed at twice the MNCC.

The virucidal effect has been assumed as the main antiherpetic activity of essential oils and their compounds, occurring by disruption of the envelope of viral particles [53,54], an event that was observed by transmission electron microscopy in HSV-1 virions treated with thymol-related monoterpenoids [55]. Nevertheless, antiviral activity can be observed in the absence of virucidal effects [56,57,58]. Similarly, in this study, *S. impressa* EO also did not show virucidal effects against HSV-1 and HSV-2 but revealed antiviral activity against both viruses.

#### 2.4.2. Effect on Virus Yield

The antiviral effect was evaluated against cultured E6 cells infected with HSV-1 and HSV-2 at a multiplicity of infections, allowing just one replication cycle. The EO was present along the infection period (26 h), having been added 0.5 h post-infection (p.i.), at concentrations ranging from 0 (control, non-treated infected cultures) to 10 μg/mL.

**Figure 8:**
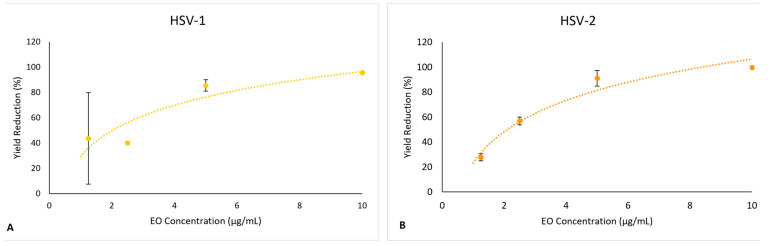
Effect of the *S. impressa* EO in the production of infectious viruses in Vero E6 cells (dots and orange trendline): (**A**) HSV-1; (**B**) HSV-2. Mean ± SD from two assays with duplicates for each virus.

*S. impressa* EO led to a reduction in the yields of both viruses when compared to infectious viral particles produced in control conditions (Medium-FBS2). This reduction was dose-dependent, with a maximum inhibitory effect of 90% at the MNCC and with IC_50_ values (50% inhibition of virus yield) of 2.54 μg/mL and 1.59 μg/mL, for HSV-1 and HSV-2, respectively (Figure 8A,B). In the positive controls for antiviral effect (treatment with acyclovir), the inhibitory effect was >99.9%.

In order to account for the toxicity of the EO against the cells, the selectivity index (SI) was calculated as the ratio between the values of CC_50_ and IC_50_, as detailed in the experimental section. A SI of 9.01 was obtained for HSV-1 and 14.4 for HSV-2. These SI values, close or higher to 10 against both viruses, are indicative that *S. impressa* EO might be considered for further evaluations as an antiherpetic agent since, according to [54], SI ≥ 4 is appropriate for an antiviral agent.

#### 2.4.3. Effect on the Virus Replication Cycle

Time-of-EO-addition assays were performed to investigate the inhibitory effect of *S. impressa* EO in the replication cycles of both viruses. These experiments involved treatments with EO at 10 mg/mL until the end of the infection (26 h p.i.) but starting at four different post-infection times (0.5 h, 3 h, 6 h, and 9 h p.i.) in replicates of infected cells and non-treated cells (controls).

**Figure 9:**
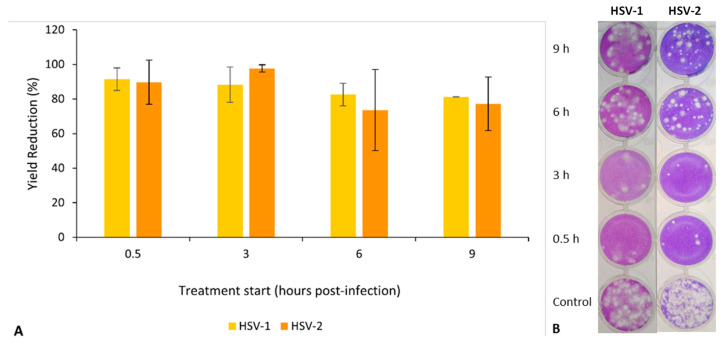
Effect of *S. impressa* EO addition time (in hours post-infection) in the production of infectious viruses (HSV-1 and HSV-2) in Vero E6 cultures, after an infection period of 26 h. (**A**) Virus yield reductions (in relation to untreated controls); mean ± SD from two assays with duplicates for each virus. No significant differences were observed. (**B**) Titration of viral progenies (dilution 10^−3^).

When limiting the time of contact with EO during virus replication, the results showed a higher inhibitory effect (more than 85% for both viruses) for treatments starting in the early period of infection (0.5 h and 3 h p.i.) and a lower inhibitory effect (under 85%, but still greater than 65%) when the treatment started later (6 h and 9 h p.i. (Figure 9A). The viral plaques observed in the wells inoculated with the dilution 10^−3^ in the titration of the different viral productions clearly evidence these results (Figure 9B).

Altogether, these results indicate an antiviral effect of *S. impressa* EO against HSV-1 and HSV-2 during their replication in infected cells. Moreover, considering the consistently negative results after direct exposure of both viruses to EO, a true effect on its replication was observed, which cannot be assigned to the virucidal effect against the new viral particles produced during the infection.

As the infection was synchronized (adsorption at 0 °C), and the EO addition only occurred 0.5 h p.i. to prevent interaction with the viral particles still present at time 0 (virus entry), the positive effect in the reduction in virus yields, combined with the absence of virucidal effect, clearly indicate an effect on virus–cell interactions These interactions are most likely to occur in more than one of the multiple steps of the replication cycle, involving a complex morphogenesis ending in the production of the enveloped viral particles as previously reviewed [59,60].

The experiments involving the addition of the *S. impressa* EO at different times corroborated the previous results, showing a higher antiviral effect when the addition occurred during the early stage of infection. However, considering the still positive effect when the EO was added during the late period of infection (6 h and 9 h p.i.), it is not possible to suggest a mode of action for the EO antiviral activity.

In *S. insularis*, the only species of *Santolina* genus in which the antiherpetic activity of the EO has been studied [7], the antiviral activity was mainly attributed to a virucidal effect and not to a direct action in the virus replication cycle, as observed with *S. impressa* EO. Further studies are necessary to understand the mode of antiviral action of *S. impressa* EO, which differs from most of the EOs and/or their compounds with antiherpetic activity previously studied.

## 3. Materials and Methods

### 3.1. Chemicals

All chemicals used in the research were reagent grade and purchased from commercial sources as follows: Glutaraldehyde; sodium phosphate buffer; acetone; ethanol; Leica Historesin; periodic acid—Schiff, toluidine blue O; Sephadex G50; dimethyl sulfoxide (DMSO); thiazolyl tetrazolium bromide (MTT); formaldehyde; crystal violet; and acyclovir were from Merck-Sigma-Aldrich (Darmstadt, Germany). CO_2_ independent medium, fetal bovine serum (inactivated), GlutaMAX, and gentamicin were from Gibco, Thermo Fisher Scientific (Waltham, MA, USA). 

### 3.2. Plant Material

Collective samples from vegetative and floral branches of *Santolina impressa* Hoffmanns. and Link (Figure 1) were randomly collected during full blossom (June/July) from natural populations occurring throughout a coastal area of Southwestern Portugal, from Comporta (38°22′ N, 8°44′ W) until Pinheiro-da-Cruz (38°14′ N, 8°44′ W), about 70 Km Southeast of Lisbon. A voucher specimen was deposited in the Herbarium of the Botanical Garden of Lisbon University under the accession number LISU: 233493.

### 3.3. Secretory Structures Characterization

#### 3.3.1. Stereomicroscopy

For a general macroscope overview, expanded leaves and disc florets from the capitula of *S. impressa* were examined under an Olympus SZH-ILLK stereomicroscope (Olympus Optical Co., Ltd., Tokyo, Japan). Images were recorded using an Olympus C-7070 Wide Zoom digital camera (Olympus Imaging Corp., Tokyo, Japan).

#### 3.3.2. Scanning Electron Microscopy (SEM)

In order to study the distribution and micromorphology of the secretory structures, samples of leaves and disc-florets from capitula at different stages of development were fixed with 2.5% glutaraldehyde in 0.1 M sodium phosphate buffer, pH 7.2, for 24–48 h at 4 °C. After washing in the same buffer, the material was dehydrated in a graded acetone series, critical point dried with CO_2_, and coated with gold. Observations were carried out on a JEOL T220 scanning electron microscope (JEOL Ltd., Tokyo, Japan) at 20 kV, and images were recorded on film black and white Kodak Tmax 100.

#### 3.3.3. Light Microscopy (LM)

For anatomical study under LM, samples of leaves and disc-florets from capitula at various developmental stages were fixed as for SEM, dehydrated through a graded ethanol series, infiltrated, and embedded in Leica Historesin^®^ following standard methods. Sections (2–5 μm thick) were cut using a Leica RM-2155 microtome (Leica Microsystems, Nussloch, Germany) and stained with periodic acid—Schiff (PAS) reagent/toluidine blue O [61] for general histology and detection of total polysaccharides and starch.

The main classes of metabolites present in the secreted material were investigated in hand-cut sections of fresh material, as referred to previously [62]. Observations were made with a Leica DM-2500 microscope (Leica Microsystems CMS GmbH, Wetzlar, Germany), and images were recorded digitally using a Leica DFC-420 camera (Leica Microsystems Ltd., Heerbrugg, Switzerland) and the Leica Application Suite software (version 2.8.1). Otherwise, observations under UV and blue wavelengths were made with a Leitz SM-LUX epifluorescence microscope (Leitz-Wetzlar, Wetzlar, Germany) equipped with an HBO 50 W mercury vapor lamp, a filter block A (excitation filter BP 340–380, dichroic mirror 450, barrier filter LP-430), and a filter block I2 (excitation filter BP 450–490, barrier filter LP-515). Images were recorded in color-positive film Kodak^®^ Provia 400 ASA.

### 3.4. Extraction and Chemical Analysis of the Essential Oil (EO)

#### 3.4.1. Essential Oil Isolation

*Santolina impressa* EO was isolated from fresh plant material by hydrodistillation for 3 h, using a Clevenger-type apparatus, according to the European Pharmacopoeia [63], with a distillation rate of 3 mL/min. The EOs were stored at −20 °C in the dark until analysis. The essential oil yield was calculated on a fresh-weight basis.

#### 3.4.2. Essential Oil Analysis and Quantification

Volatiles were analyzed by gas chromatography with a flame ionization detector (GC-FID) for component quantification and gas chromatography coupled to mass spectrometry (GC-MS) for component identification. 

The GC-FID analysis was performed on a Perkin Elmer GC 8700 gas chromatograph equipped with two flame ionization detectors, with a data handling system and a vaporizing injector port into which two columns of different polarities: a DB-1 fused-silica column (100% dimethylpolysiloxane, 30 m × 0.25 mm i.d., film thickness 0.25 µm; J & W Scientific Inc., Folsom, CA, USA) and a DB-17HT fused-silica column ((50% phenyl)-methylpolysiloxane, 30 m × 0.25 mm i.d., film thickness 0.15 µm; J & W Scientific). The oven temperature was programmed from 45 to 175 °C, at 3 °C/min, then up to 300 °C at 15 °C/min, and held isothermal for 10 min, for a total run time of 61.67 min. Gas chromatographic settings were as follows: injector and detector temperatures were 280 °C and 290 °C, respectively; carrier gas was H_2_ at 30 cm/s. The EOs percentage composition was determined by integration of the peak areas without the use of correction factors, in accordance with ISO 7609 [64]. The values shown represent the mean value of two injections.

The GC-MS unit consisted of a Perkin Elmer Clarus 600 gas chromatograph, equipped with DB-1 fused-silica column (100% dimethylpolysiloxane, 30 m × 0.25 mm i.d., film thickness 0.25 µm; J & W Scientific), and interfaced with a Perkin-Elmer Clarus 600T mass spectrometer (software version 5.4.2.1617, Perkin Elmer, Shelton, CT, USA). Injector and oven temperatures were as above; transfer line temperature, 280 °C; ion source temperature, 220 °C; carrier gas, helium, adjusted to a linear velocity of 30 cm/s; split ratio, 1:40; ionization energy, 70 eV; scan range, 40–300 u; scan time, 1 s. The identity of the components was assigned by comparison of their retention indices (RI) relative to C_9_–C_17_ *n*-alkane indices and GC-MS spectra from a laboratory-made library based upon the analyses of reference EOs, laboratory-synthesized components, and commercially available standards.

### 3.5. Antiviral Activity of the Essential Oil

#### 3.5.1. Cells and Viruses

The antiviral activity was evaluated against herpes simplex virus type 1 (HSV-1) (strain SC 16) and HSV-2 (strain HD) in cultured Vero E6 cells (ATCC, reference CRL-1586). Cells and viruses were kindly provided by Instituto Nacional de Saúde Dr. Ricardo Jorge (INSA, Lisboa, Portugal).

The cells were routinely cultivated at 37 °C in T25 flasks containing Medium-FBS10, which consisted of CO_2_ Independent Medium supplemented with 2 mM GlutaMAX, 0.5 mg/mL gentamicin, and 10% fetal bovine serum (inactivated) (FBS). Both viruses were produced by infecting sub-confluent cultures with a multiplicity of infection (MOI) of 0.1 PFU (plaque-forming units) per cell in Medium-FBS2 (2% FBS). After visualization of the complete cytopathic effect (CPE) (i.e., a suspension of detached cells), the medium was clarified by low-speed centrifugation (3000× *g*, for 5 min) to recover the viruses in suspension, removing the pelleted cells and cell debris. Virus suspensions were kept at 4 °C or −80 °C (for long-term storage).

Virus titrations were carried out by plaque reduction assays, applying serial dilutions of the viral suspensions in Medium-FBS2 to sub-confluent cell cultures in 24-well plates (0.1 mL/well). After an adsorption period of 1 h, Medium-FBS2 supplemented with 2% sterile Sephadex G50 was added (0.5 mL/well), and the plates were kept immobilized at 37 °C under a humid atmosphere during the titration period (3 days). Later, the cells were fixed with 10% formaldehyde for 30 min, washed with tap water, and stained with 0.2% crystal violet for 15 min. After washing again to remove the stain, the plates were air-dried, and the viral plaques were counted.

#### 3.5.2. Essential Oil Stock and Work Solutions

EO stock and work solutions were prepared at 180 mg/mL and 600 µg/mL in Dimethyl sulfoxide (DMSO) and kept at −20 °C. 

#### 3.5.3. Cytotoxicity Evaluation

In order to determine the EO cytotoxicity, cells were seeded into 96-well plates and incubated at 37 °C with Medium-FBS10 until sub-confluency; then, the culture medium was replaced by 100 µL of Medium-FBS2 with different EO concentrations (1–80 µg/mL) or Medium-FBS2 (controls), at least two assays with four replicates for each concentration. Afterward, the plates were incubated at 37 °C for 24 h, and subsequently, the cells’ viability was determined by the thiazolyl tetrazolium bromide (MTT) assay [65]. The maximum non-cytotoxic concentration (MNCC) and the concentration inducing 50% toxicity (CC_50_) were calculated.

#### 3.5.4. Antiviral Assays

*Virucidal Effect*. Viral suspensions of 100 μL, with ~10^6^ PFU, were incubated with non-cytotoxic concentrations of EO (control samples with the same concentration of DMSO), for 2 h, at room temperature with stirring. After the treatment, each viral suspension was titrated. Virus inactivation was determined by comparison of the titters of treated and control samples.

*Effect on Virus Yield*. Sub confluent cell monolayers, grown in 6-well plates, were infected with each virus at a multiplicity of infection (MOI) = 3 PFU/cell (two assays with duplicates for each virus). After an adsorption period of 1 h at 4 °C, the viral inoculum was removed, and 2 mL of pre-warmed Medium-FBS2 (37 °C) was added (time 0 of infection). Thirty minutes later, the medium was replaced by Medium-FBS2 with different concentrations of EO (0 (controls) to 10 µg/mL) and acyclovir at 10 µg/mL (positive control of treatment), maintaining the plates at 37 °C for 26 h. The supernatants of the cultures containing HSV-1 or HSV-2 virions were then collected and titrated as described in Section 3.5.1.

The titters of the viral suspensions produced under treatment were compared to controls. Virus yield reductions (in percentage) were calculated, considering that 100% corresponds to the titter of the virus produced in control (non-treated) conditions. The EO concentration leading to a reduction of 50% in virus yield (IC_50_) was determined, as well as the selectivity index (SI), which is the ratio between the values of CC_50_ and IC_50_: CC_50_/IC_50_. 

*Effect on the Replication Cycle*. To detect the steps of the replication cycle affected, the EO was assayed at 10 µg/mL, in experiments only differing from the previously described (Section 3.5.2), in the EO addition times: 30 min, 3-, 6-, and 9 h p.i. Two assays with duplicates for each virus were carried out.

#### 3.5.5. Data Analysis

Regression analysis of dose-response curves was used for EO cytotoxicity determination and effect on virus yield evaluation. The results were expressed as average ± standard deviation. All statistical analysis was performed using Microsoft Excel 2010.

## 4. Conclusions

*Santolina impressa* is an aromatic Asteraceae species endemic to Portugal, with a limited geographical distribution, that is included in a complex taxonomic aggregate. The present study provides, for the first time, a detailed characterization of *S. impressa* secretory structures and of their pattern of distribution that can contribute to a better taxonomic delimitation of this species. The leaf morpho-anatomical traits described here correlate well with the environmental conditions of the coastal sand dune ecosystem that distinguishes this species’ habitat. Essential oil characterization revealed a composition like the previously reported for this species growing wild in Portugal. The antiherpetic activity of the EO against HSV-1 and HSV-2, in the absence of virucidal effect, is a promising result that requires further studies to investigate its mode of action in the replication cycle and to ascertain which compounds and/or compounds enantiomeric forms, are involved in the observed antiviral activity.

## Figures and Tables

**Table 1:** Results of the histochemical tests used for the characterization of the main classes of compounds present in the secretion of the glandular trichomes and secretory ducts of *Santolina impressa* leaves.

Target Class of Compounds	Histochemical Tests	Positive Reaction Color	Glandular Trichomes	Secretory Ducts
Total lipids	Sudan Black B	Dark blue to Black	++	++
	Sudan Red IV	Red	++	++
Acidic/Neutral lipids	Nile Blue A	Blue/Red	++ (Red)	++ (Red)
Unsaturated lipids	Osmium Tetroxide	Black	++	++
EOs and resins	Nadi Reagent	Blue (EOs)/Red (Resins)	++ (Violet)	++ (Red)
Polysaccharides	PAS	Bright rose	−	−
Pectins	Ruthenium Red	Red	−	−
Phenolic compounds	Potassium Dichromate	Brown-orange	++	++
	Ferric Trichloride	Dark brown	++	++
Flavonoids	Aluminum Chloride (UV)	Green yellowish	++	++

++ strong positive reaction; − negative reaction.

**Table 2:** Percentage composition of the essential oil isolated flowering aerial parts of *Santolina impressa*.

Components	RI	*Santolina impressa*
Tricyclene	921	0.1
α-Thujene	924	0.4
α-Pinene	930	1.7
Camphene	938	1.5
Thuja-2,4(10)-diene *	940	0.2
Sabinene	958	1.2
β-Pinene	963	12.6
Dehydro-1,8-cineole	973	0.1
β-Myrcene	975	6.0
Yomogi alcohol	978	8.8
α-Phellandrene	995	0.3
α-Terpinene	1002	0.8
*p*-Cymene	1003	0.2
1,8-Cineole	1005	6.2
β-Phellandrene	1005	10.4
Limonene	1009	8.1
*cis*-β-Ocimene	1017	1.3
*trans*-β-Ocimene	1027	1.1
γ-Terpinene	1035	1.2
Artemisia alcohol	1055	2.1
Terpinolene	1064	0.7
Dehydro sabina ketone *	1066 *	0.1
2-Methyl butyric acid isoamyl ester	1074	0.2
Isopentyl isovalerate	1084	0.1
*trans*-*p*-2-Menthen-1-ol	1099	0.3
Camphor	1102	7.1
*trans*-Pinocarveol	1106	0.3
*cis*-*p*-2-menthen-1-ol	1110	0.2
Pinocarvone	1121	0.3
α-Phellandrol *	1134	1.1
Borneol	1134	1.1
Terpinen-4-ol	1148	2.8
α-Terpineol	1159	1.0
Verbenone	1164	1.9
Piperitone	1211	0.4
Bornyl acetate	1265	0.5
Lavandulyl acetate	1278	t
Geranyl acetate	1370	t
β-Caryophyllene	1414	0.2
*allo*-Aromadendrene	1456	0.1
*ar*-Curcumene	1474	0.8
γ-Muurolene	1469	0.2
Germacrene D	1474	0.4
Bicyclogermacrene	1487	0.5
δ-Cadinene	1505	0.2
Spathulenol	1551	0.7
β-Caryophyllene oxide	1561	0.2
Anhydrooplopanone	1576	0.4
T-Cadinol	1616	0.2
α-Cadinol	1626	0.2
**% Identification**		86.5
**Grouped components**		
Monoterpene hydrocarbons		47.8
Oxygen-containing monoterpenes		34.3
Sesquiterpene hydrocarbons		2.4
Oxygen-containing sesquiterpenes		1.7
Others		0.3

RI—Retention index relative to C_9_–C_17_ *n*-alkanes on the DB-1 column; * Identification based on mass spectra only; t—traces (<0.05%).

## Data Availability

Not applicable.
